# Ki67 assessment in invasive luminal breast cancer: a comparative study between different scoring methods

**DOI:** 10.1111/his.14781

**Published:** 2022-09-19

**Authors:** Ayat Lashen, Michael S Toss, Andrew R Green, Nigel P Mongan, Emad Rakha

**Affiliations:** ^1^ Academic Unit for Translational Medical Sciences, School of Medicine University of Nottingham Nottingham UK; ^2^ Department of Pathology, Faculty of Medicine Menoufia University Shebin El Kom Egypt; ^3^ Nottingham Breast Cancer Research Centre University of Nottingham Nottingham UK; ^4^ Department of Histopathology Sheffield Teaching Hospitals NHS Foundation Trust Sheffield UK; ^5^ School of Veterinary Medicine and Sciences University of Nottingham Nottingham UK; ^6^ Department of Pharmacology Weill Cornell Medicine New York USA

**Keywords:** assessment, breast cancer, hotspot, Ki67, methods

## Abstract

**Background:**

Ki67 reflects the proliferation activity in breast cancer (BC). However, an optimal method for its assessment in clinical settings has yet to be robustly defined. In this study we compared several methods to score Ki67 to identify a reliable and reproducible method for routine practice.

**Methods:**

Sections from luminal BC cohort (*n* = 1662) were immunohistochemically stained with Ki67 and were assessed for the percentage, pattern, and intensity of expression. Ki67 positivity was evaluated using three methods: (i) quantification of Ki67‐positive cells among 1000 invasive tumour cells within hotspot, (ii) average estimation of Ki67 within a defined hotspot, and (iii) average estimation of Ki67 positivity within the whole section. Time required for scoring, interobserver agreement and association with outcome were determined.

**Results:**

The mean percentage of Ki67 expression per 1000 cells method was 16%, while the mean value of Ki67 scores using the average estimation within hotspot and whole slide were 14% and 12%, respectively. Quantification of Ki67‐positive cells within 1000 cells had the highest degree of consistency between observers, and the highest hazard ratio predicting patient outcome when compared to using different common Ki67 cutoffs, which was independent of the other two methods. Granular pattern of Ki67 expression was associated with poorer outcome as compared to the other patterns.

**Conclusion:**

Assessment of Ki67 expression using quantification positive cells among 1000 tumour cells is an optimal method to achieve high reliability and reproducibility. Comment on the predominant Ki67 expression pattern would add prognostic and predictive value in luminal BC.

## Graphical abstract

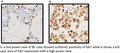

## Introduction

Cellular proliferation is an important indicator of tumour behaviour and prognosis. The proliferation index as assessed by Ki67 immunohistochemical (IHC) staining in histological tissue sections is used as a surrogate marker for cellular proliferation.^1^, ^2^ In invasive breast cancer (BC), the Ki67 labelling index is an essential tool used to classify luminal (oestrogen receptor [ER]‐positive) tumours into two IHC surrogate subclasses: luminal A and B, which have different outcomes and therapeutic approaches.^3^, ^4^ Although Ki67 assessment is used primarily as a prognostic factor and for management plans in BC, it also has predictive value for adjuvant therapy response and treatment efficacy.^5^

Ki67 is a nuclear protein that is expressed during all cell cycle phases except G0.^6^, ^7^ Visual assessment of Ki67 immunohistochemically stained BC tissue sections is considered the standard method for Ki67 evaluation, but this approach is time‐ and cost‐intensive.^8^ Ki67 is expressed mainly in the nuclei of the proliferating cells or demarcates the chromatin spindles of the cells in the mitotic phase. Despite the presence of several methods for Ki67, there is a consensus that any nuclear and/or mitotic spindle staining should be considered in the final score regardless the intensity or the pattern of the staining.^9^ Occasional cytoplasmic and/or cell membrane staining of Ki67 can be observed but are not included in the Ki67 assessment.^8^ Staining usually has several intensities ranging from faint to strong, and various patterns including homogenous, coarse‐granular, fine granular/dotted, nuclear membrane, subnuclear membrane condensations, nucleolar, and a mixture of all the aforementioned patterns, which occurs in the majority of cases.

Various methods of Ki67 scoring have been proposed; however, no systematised or standardised methodology has been adopted to date.^10^, ^11^ These include Ki67 assessment within tumour hotspots, defined as the area within an invasive tumour with the most Ki67‐positive cells by counting Ki67‐positive cells within a fixed number of tumour cells and calculate the percentage of positivity in the hotspot^12^; average expression of Ki67 expression within the whole tumour by scanning the whole slide at low power and estimating the percentage of positivity.^13^ Another protocol utilises a visual estimate of Ki67 through a five‐grade scale, where tumour sections are qualitatively graded, and pathologists estimate the ratio of positive/negative cells in a simple 5‐grade scale.^14^ The International Ki67 Breast Cancer Working Group (IKWG) recently recommended a standardised visual scoring method using an online scoring application,^2^ whereas others suggested other methods including counting the average by quantifying 1000 cells in multiple random selected areas regardless of the Ki67 hotspot.^15^

Although there is little doubt that Ki67 is a key prognostic and predictive biomarker in BC, there remains concern regarding the optimal way for its assessment in routine clinical practice. The absence of a standardised method of Ki67 assessment has contributed to challenges in the consistency, reproducibility, and reliability of Ki67 scoring. In addition, the optimal cutoff for dichotomisation low versus high Ki67 groups is still undefined and it is greatly varied in the previous literature.^16^, ^17^, ^18^ For this reason, here we set out to robustly compare between various methods of visual Ki67 assessment while considering practicality, time effectiveness, reproducibility, the prognostic and predictive value, heterogeneity, and the biological value of different staining intensities, patterns and sublocalisations utilising a large cohort of luminal ER‐positive and human epidermal growth factor receptor (HER2)‐negative BC to identify the optimal scoring method that can be clinically valid and applicable.

## Materials and methods

This study was conducted on a large cohort (*n* = 1662) of ER‐positive, HER2‐negative BC with Nottingham lymph node (LN) stage 1 (negative LNs) or stage 2 (tumour metastasis in 1–3 regional LNs). Detailed clinicopathological parameters were available for 1583 patients and are summarised in Supplementary Table S1. Outcome data were available, including BC‐specific survival (BCSS) defined as the time (in months) from the date at diagnosis to the time of last date that patient was known to be alive or date of BC‐related death, and distant metastasis‐free survival (DMFS), defined as the time (in months) from the date at diagnosis until the first event of distant metastasis. Data on ER, progesterone receptor (PR), and HER2 were derived from the original pathology reports. Endocrine therapy was offered to 90% of the patients, while 17% received chemotherapy. None of the patients included in the study received neoadjuvant therapy. At least 3–4 haematoxylin and eosin (H&E)‐stained sections from each case were histologically reviewed to select one representative section that contained the highest tumour burden and histological grade for assessment of cellular proliferation.

### Ki67 immunohistochemical staining

Full‐face sections, from the resection specimens of the study cohort, were prepared from the selected formalin‐fixed paraffin‐embedded (FFPE) tissue blocks. The IHC staining was performed automatically using clinically‐validated, the DAKO Cytomation EnVision+ detection system (Glostrup, Denmark) according to the standard protocols. Briefly, sections were deparaffinised, rehydrated, and microwaved in citrate buffer (pH 6.0) and used for the antigen retrieval. Primary antibody (anti‐human Ki67 monoclonal antibody MIB1, DAKO) was applied and incubated for 30 min. Positive tissue control of normal tonsil was included in each staining run, while a negative control was included by omitting the primary antibody.

### Ki67 scoring

Ki67 was assessed using visual examination of the stained sections using a light microscope (ECLIPSE Ni‐U; Nikon Instruments, Tokyo, Japan) with 10× ocular subjective lens (eyepieces) and 40× objective lens with a wide field diameter of 0.63 mm. Invasive tumour cells only were assessed, while areas of necrosis, poor section quality, inviable tissue, and positivity within ductal carcinoma *in situ* (DCIS) or inflammatory cells were excluded.^19^ Sections were evaluated, and the percentage of positivity of Ki67 expression was assessed using the following methods:

1. Quantification of Ki67‐positive cells per fixed number of tumour cells (*n* = 1000 cells) within the hotspot and the percentage was calculated.^12^, ^20^ A hotspot was defined as the area with the highest density of Ki67‐positive tumour cells, identified at 10× magnification, compared to the surrounding tumour tissue areas and then 40× magnification was used to score Ki67. The number of fields to count Ki67 positivity among 1000 tumour cells depended on the cellularity. In cellular tumours with minimal intervening stroma, high‐power field (HPF) had 500–1000 cells so 1–2 field areas were sufficient for counting, whereas in the less cellular tumours, HPF may have 200–400 cells, so more field areas are needed to count 1000 cells.
2. Average estimation of Ki67 expression within the hotspot. The average percentage of Ki67‐positive tumour cells, in relation to all tumour cells within the hotspot, was subjectively (without cellular count) estimated in one field at 20× magnification.
3. Average Ki67 expression within the whole slide. Average estimation of the proportion of Ki67‐positive cells in the whole tumour area including hot and cold spots areas was carried out. The whole slide was scanned at low power (5× or 10×) magnification and then the mean percentage of Ki67‐positive cells was assessed at multiple high‐power views covering the different areas of the tumour.

**Figure 1:**
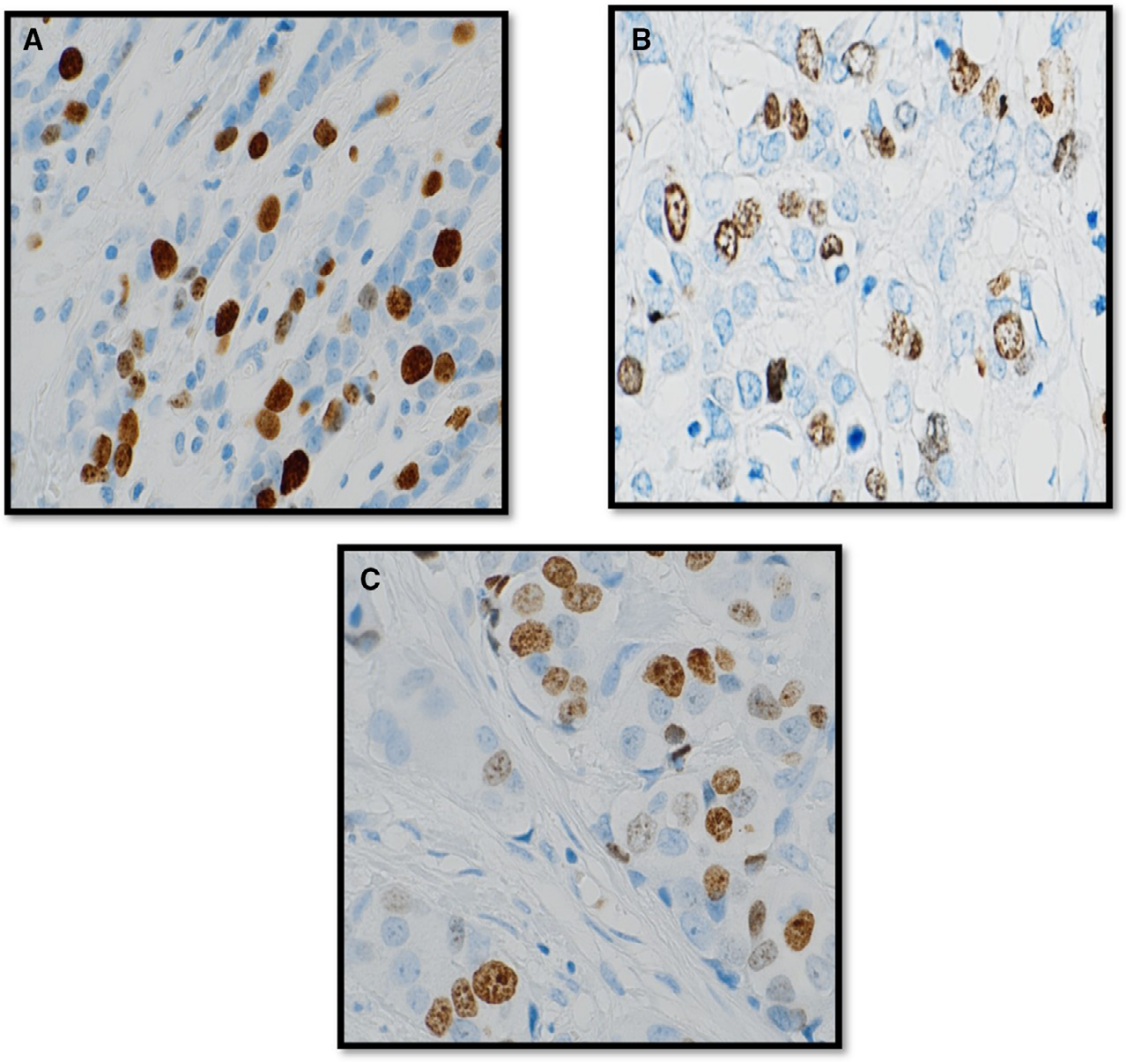
Different patterns of Ki67 staining. (A) Homogenous staining of the nucleoplasm. (**B**) Granular pattern showed either stained nucleoli or granules of distinct size dispersed throughout the nucleoplasm. (**C**) Mixed pattern of homogenous and granular ones.

We also evaluated the intensity and pattern of Ki67 expression. The intensity was classified into strong, moderate, or weak staining compared to the intensity within the positive tissue control. The staining pattern (at the cellular level) was classified into: 1, homogenous (uniform) staining of the nucleoplasm, 2, granular pattern, which refers either to stained nucleoli or granules of distinct size dispersed throughout the nucleoplasm, and 3, a mixed pattern that is strongly stained granules against a homogenously fainter stained background^21^, ^22^ (Figure 1). Finally, a combined score of the various intensities and percentage of Ki67 expression was used to estimate the Histo score (H‐score) of Ki67 to achieve a numerical continuous score ranging between 0 and 300^23^ and the Quick score (the sum of the score for the proportion of cells stained and the score for the intensity of staining) to get the total score, range of 0–8.^24^ The scoring time, in minutes, was recorded for each method. In addition, multiple common cutoffs that were previously described to categorise Ki67 into distinct high and low proliferating tumours including 10%,^25^, ^26^ 14%,^17^ 20%,^27^ and 30%^2^ were used to compare the performance of different scoring methods.

The whole cohort was assessed using these methods by an experienced pathologist with more than 5 years experience and under supervision by a breast histopathology consultant. A second experienced pathologist independently scored 25% of the cohort and the level of interobserver agreement was analysed to assess the degree of interobserver agreement. These cases were chosen randomly to avoid bias.

### Assessment of the clinical impact of Ki67 intratumoural heterogeneity on BC

To assess the heterogeneity of Ki67 staining objectively, the difference (Δ Ki67) in Ki67 expression between the quantification of Ki67 in hotspot and average Ki67 in the whole slide was calculated,^12^, ^19^ defined here as the heterogeneity index, which was used to dichotomise the cohort into two groups, low versus high Ki67 heterogeneity index, based on the median and correlation with clinicopathologic parameters and outcome data was carried out.

### Statistical analysis

Statistical Package for the Social Sciences software v. 26.0 (SPSS, Chicago, IL, USA) was used for the statistical analysis. In addition to the previously described cutoffs for Ki67 proliferation index groups, Ki67 percentages of positivity were categorised based on the median,^18^, ^28^ which were 12%, 10%, 10% in the quantification method, average estimation within hotspot, and the average in whole‐slide methods, respectively. For statistical purposes, the pattern of Ki67 expression was categorised into granular and other types. Also, the H‐score of Ki67 expression was categorised into low and high groups based on the median (H‐score 50), while the Quick score was divided into a low proliferation group ≤4 and high proliferation group >4. The interclass correlation coefficient test was used to assess concordance among observers. The Chi‐square test was used for analysis of the categorical data, while Spearman's rank correlation was used to correlate for continuous variables. The outcome analysis was assessed using Kaplan–Meier curves and the log‐rank test. The univariate and multivariate Cox Regression model were also applied. For all tests, *P* < 0.05 (two‐tailed) was considered statistically significant.

## Results

### Interobserver agreement of Ki67 expression assessment

There was consistent agreement between observers regarding Ki67 assessment methods with Kappa values of 0.9 and 0.8 obtained for Ki67 quantification in hotspot and average estimation in the hotspot, respectively, whereas evaluation of average Ki67 scoring per the whole section had a Kappa value of 0.7. There was also a strong positive correlation between the quantification method and the average estimation method within the hotspot (*r* = 0.8, *P* < 0.0001). Also, it was observed between the quantification method and the average in the whole slide (*r* = 0.9, *P* < 0.0001).

### Distribution of Ki67 expression in BC with the different scoring methods

The mean of Ki67 percentage using the quantification method per 1000 cells was 16% (range 1–98%), while the mean of the Ki67 scores with estimation of the average within hotspot was 14% (range 1–95%). Ki67 proportion in average of the whole slide showed a mean of 12% (range 1–95%). Supplementary Figure S1 shows examples of Ki67 expression in two different BC cases demonstrating the low and high expressions levels. When Ki67 was dichotomised using different cutoffs, the percentage of high proliferating tumours was higher in the quantification method than both average methods. For instance, using 14% as a cutoff, there was 41% of cases with a high proliferation rate using the quantification method compared to 33% of cases in the average evaluation of Ki67 in the whole slide (Supplementary Table S2).

In all, 82% of the cohort showed homogeneous and a mixed pattern of Ki67 staining, while 18% showed a pure granular pattern. Regarding the Ki67 intensity, 38% of the cases showed strong expression, whereas 62% showed moderate or weak expressions. When the percentage of Ki67 expression was combined with the intensity of staining, the low H‐score ≤50 was observed in 87% of cases. 36% of the study cases showed a low Quick score.

### Scoring time

The mean ± SD of scoring time for quantification with the 1000 cells method of Ki67 was 6.6 ± 1.2 min (median = 7 min, range 5–12 min). At the beginning of the study, quantification of 1000 cells took around 12 min, then after training and getting more experience in counting, the process took less time. The mean ± SD of time for average estimation in the hotspot were 2 ± 0.7 min, while the mean ± SD of subjective estimation in the whole slide was 1.2 ± 0.6 min. The difference between the time of Ki67 scoring with the quantification method and the average estimation in the hotspot method was statistically significant (*P* = 0.002).

**Figure 2:**
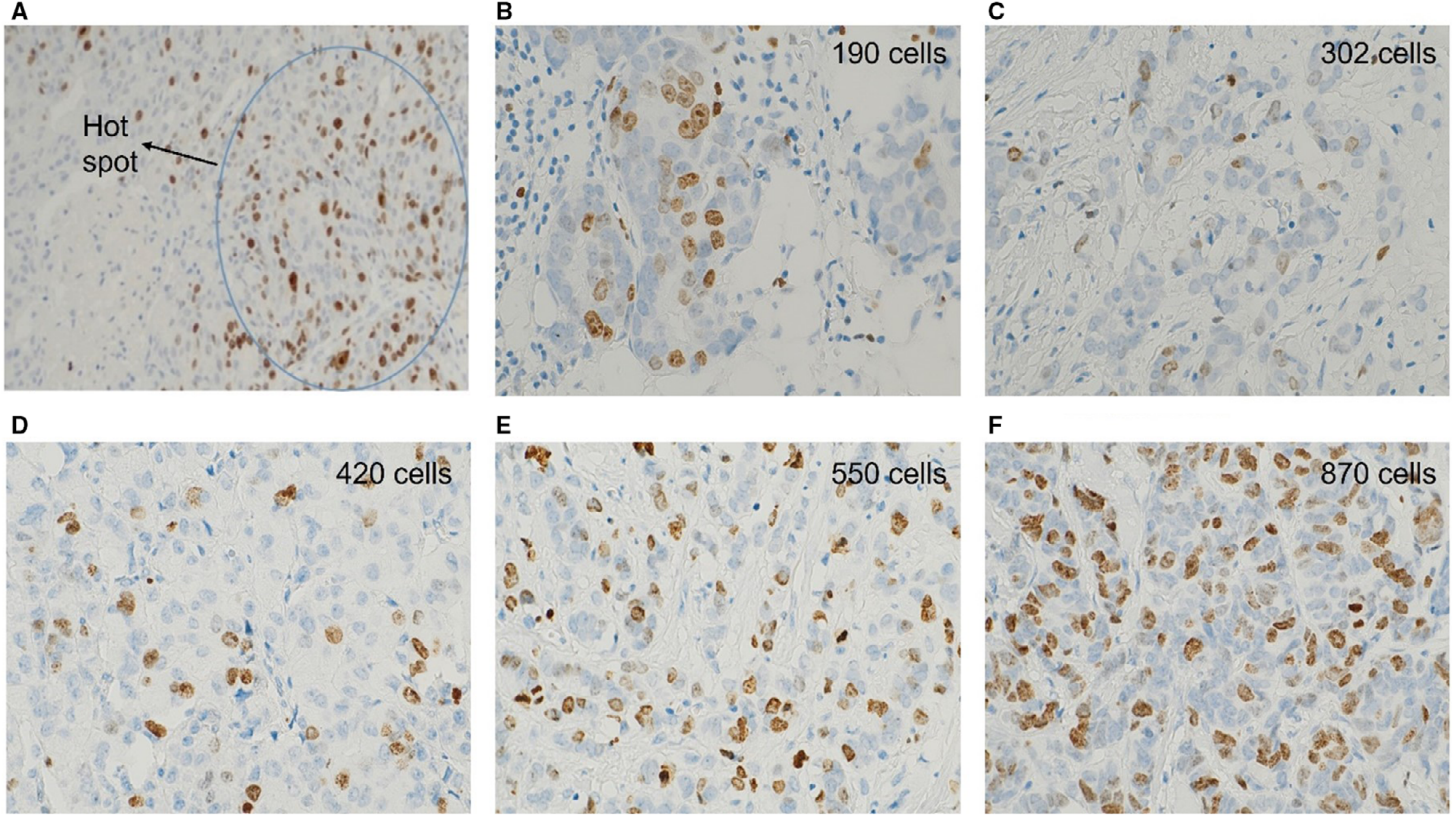
Assessment of Ki67 in clinical practice: First, pathologists need to choose hotspot area at 10× (**A**). After calibration and training, pathologists can calibrate their high‐power field (HPF) and estimate the number of cells per HPF in tumours with different cell density and assess the approximate number of HPFs that are required to count 1000 cells per case. Then they can estimate the average percentage of Ki67‐positive cells in the 1000 cells, eyeballing without an actual count of the cells. (**B–F**) Examples of different tumours densities, which affect the number of fields to count 1000 cells either in 1 HPF in very high‐density tumour or in more than 5 HPFs in low‐density tumour. This method would be more objective, with standardisation of the 1000 cells. It will also be a time‐saving task (2–3 min per case).

In clinical settings, such a counting method can be considered time‐consuming. Therefore, we considered the tumour cell density and the number of cells per specific areas in tumours with variable cellularity, aiming to reduce the time needed for the actual cell count of every case. To test the reliability of this simplified method, we scored 100 cases after training and adjustment of the range of cells per HPF in tumours with variable cells density. This reduced the time to 1–3 min per case and showed high concordance with the actual count method (Kappa = 0.9, Figure 2).

### Correlation between the Ki67 scores and the clinicopathological parameters

There was a significant association between high Ki67 scores (>12%) using quantification of cells in hotspot and younger age at diagnosis, larger tumour size, grade 3 tumours, and poor Nottingham prognostic index (NPI) (all, *P* < 0.0001). Similar findings were observed regarding Ki67 scores using estimation of the Ki67 average within hotspot and the average in the whole slide (Table 1).

**Table 1:** Relationship between Ki67 expression in luminal breast cancer assessed by various methods and clinicopathological parameters

Variables	Number (%)	Objective counting in hotspot		X^2^ *P*‐value	Subjective estimation within hot spot		X^2^ *P*‐value	Subjective estimation in whole slide		X^2^ *P*‐value
		Low (*n*, %)	High (*n*, %)		Low (*n*, %)	High (*n*, %)		Low (*n*, %)	High (*n*, %)	
Age at diagnosis (years)										
<50	390 (25)	189 (49)	201 (51)	21.5	172 (44)	218 (56)	34.3	164 (42)	226 (58)	23.8
≥50	1193 (75)	737 (62)	456 (38)	**<0.0001**	728 (61)	465 (39)	**<0.0001**	671 (56)	522 (47)	**<0.0001**
Menopausal state										
Premenopausal	452 (29)	228 (50)	224 (50)	16.9	210 (46)	242 (54)	28.0	196 (43)	256 (57)	22.4
Postmenopausal	1131 (71)	698 (62)	433 (38)	**<0.0001**	690 (61)	441 (39)	**<0.0001**	639 (57)	433 (43)	**<0.0001**
Tumour size (cm)										
≤2	1054 (67)	676 (64)	378 (36)	41.3	663 (63)	391 (37)	47.0	613 (58)	441 (42)	37.1
>2	529 (33)	250 (47)	279 (53)	**<00001**	237 (45)	292 (55)	**<00001**	222 (42)	307 (58)	**<00001**
Tumour grade										
Grade 1	358 (23)	313 (87)	45 (13)		301 (84)	57 (16)		286 (80)	72 (20)	
Grade 2	812 (51)	525 (65)	287 (35)	371.0	515 (66)	297 (37)	347.0	474 (58)	338 (42)	314
Grade 3	413 (26)	88 (15)	325 (85)	**<0.0001**	84 (20)	329 (80)	**<0.0001**	75 (18)	338 (82)	**<0.0001**
Histologic tumour types										
No special type (NST)	836 (53)	392 (47)	444 (53)		385 (46)	451 (54)		347 (42)	489 (48)	108.0
Lobular	187 (12)	129 (69)	58 (31)	129.2	128 (68)	59 (32)		123 (66)	64 (34)	
Other special types	334 (21)	273 (82)	61 (18)	**<0.0001**	254 (76)	80 (24)	101.0	243 (73)	91 (27)	
NST mixed	226 (14)	132 (58)	94 (42)		133 (59)	93 (41)	**<0.0001**	122 (54)	104 (46)	**<0.0001**
Lymph node invasion										
Absent	1098 (69)	679 (56)	419 (44)	16.5	660 (60)	438 (40)	15.5	611 (56)	487 (44)	12.1
Present	485 (31)	247 (51)	238 (49)	**<0.0001**	240 (49)	245 (51)	**<0.0001**	224 (46)	261 (54)	**<0.0001**
Lymphovascular invasion										
Absent	1246 (79)	782 (63)	464 (37)	43.8	770 (62)	476 (38)	58.1	711 (47)	535 (53)	43.7
Present	337 (21)	144 (43)	193 (57)	**<0.0001**	130 (39)	207 (61)	**<0.0001**	124 (37)	213 (63)	**<0.0001**
Nottingham prognostic index										
Good prognostic group	765 (48)	585 (76)	180 (24)		573 (75)	192 (25)		527 (31)	238 (69)	
Moderate prognostic group	721 (46)	320 (44)	401 (56)	215.2	307 (43)	414 (57)	213.4	294 (41)	427 (59)	178.6
Poor prognostic group	97 (6)	21 (22)	76 (78)	**<0.0001**	20 (21)	77 (79)	**<0.0001**	14 (14)	83 (86)	**<0.0001**

Significant *P* values are in bold.

Furthermore, a granular pattern of staining showed significant associations with high tumour grade, NST tumour type, LVI, and poor NPI (*P* = 0.009). A high quick score had significant associations with high grade and NST tumour type. Additionally, a high Ki67 H‐score showed strong associations with larger tumour size, grade 3 tumours, NST tumour type LVI, lymph node metastasis, and poor NPI (all, *P* < 0.0001).

### Outcome analysis

**Figure 3:**
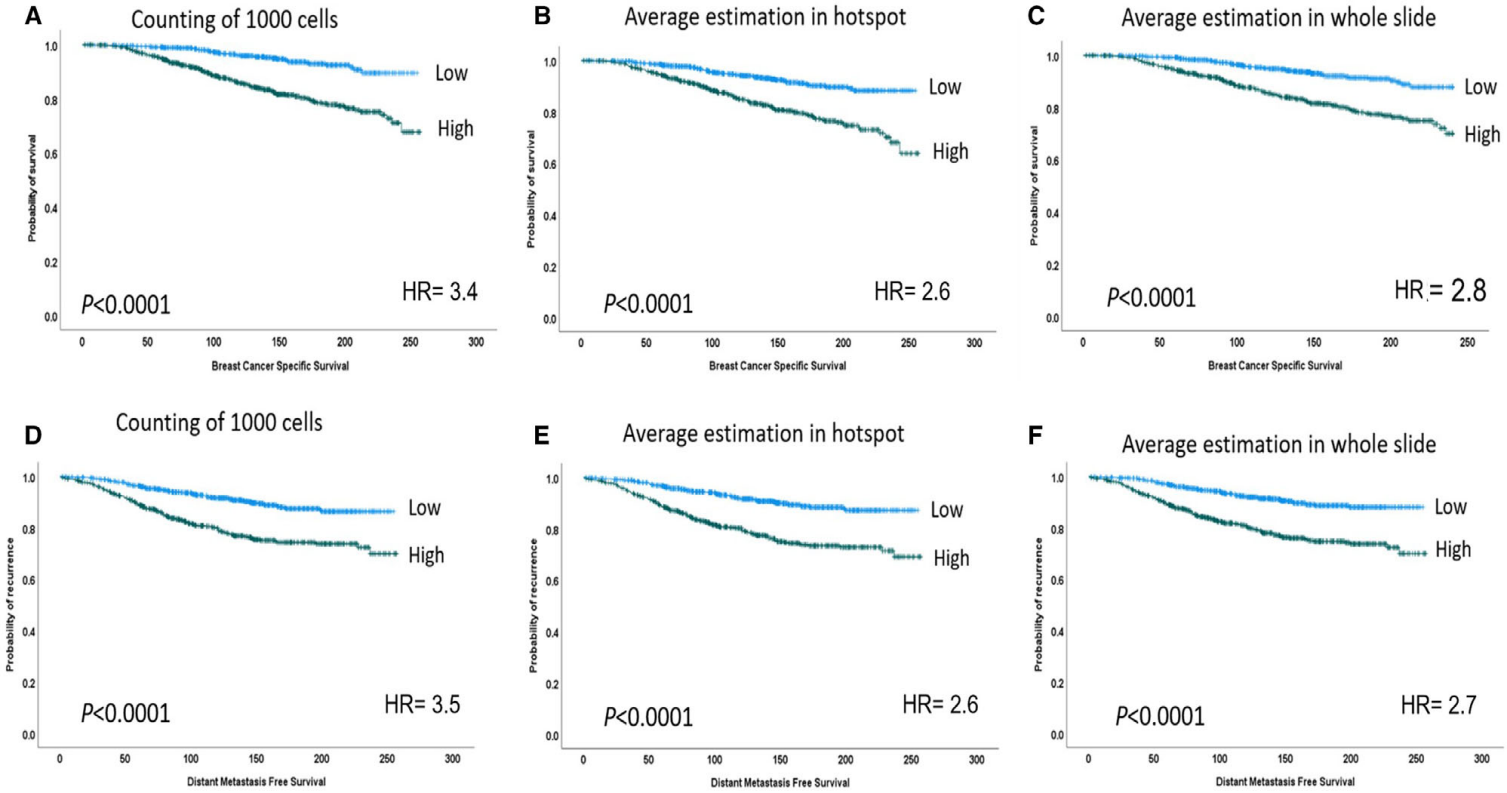
Kaplan–Meier plots showing associations of breast cancer‐specific survival with all Ki67 scoring methods (**A–C**) and the same findings were observed in terms of distant metastasis‐free survival (**D–F**).

In univariate analysis, Ki67 expression showed a significant association with outcome in terms of BCSS and DMFS (Figure 3) in all scoring methods used (all, *P* < 0.0001). However, the proportion of Ki67‐positive cells within 1000 tumour cells showed, consistently, the highest prediction of poor outcome (highest hazard ratio [HR]) and the lowest *P* value regardless of the cutoff used to define a high and low proliferation index (Table 2).

**Table 2:** Univariate Cox regression analysis for predictors of luminal breast cancer‐specific survival according to the different methods of Ki67 assessment with different Ki67 cutoffs

Cutoff Scoring Method	10%			14%			20%		30%			
	HR	95% CI	*P*‐value	HR	95% CI	*P*‐value	HR	95% CI	*P*‐value	HR	95% CI	*P*‐value
Counting Ki67‐positive cells per 1000 invasive tumour cells	3.4	2.4–4.8	**1.2 × 10** ^**−11**^	2.8	2.1–3.7	**2.7 × 10** ^**−11**^	2.4	1.8–3.1	**7.6 × 10** ^**−9**^	2.5	1.7–3.5	**0.000001**
Average estimation within the hotspot	2.6	1.9–3.6	**6.5 × 10** ^**−10**^	2.1	1.5–2.8	**9.8 × 10** ^**−7**^	2.1	1.6–2.9	**0.000003**	2.3	1.5–3.3	**0.00006**
Average estimation in whole slide	2.8	2.0–3.8	**1.6 × 10** ^**−10**^	2.7	2.0–3.6	**1.7 × 10** ^**−11**^	2.2	1.6–2.9	**0.000001**	1.9	1.2–3.1	**0.007**

HR, hazard ratio; CI, confidence interval.

Significant *P* values are in bold.

Similarly, in multivariate analysis the hotspot quantification of Ki67‐positive cells among 1000 tumour cells provided a statistically significant, prognostic value independent of the other two methods in all cutoffs applied to categorise the scores. The other two methods were statistically insignificant except for the average estimation of Ki67 within the hotspot, which showed a weak independent association with BCSS (HR = 1.6, 95% confidence interval [CI] 1.1–2.4, *P* = 0.018) when the 10% cutoff was used, and lost significance when other cutoffs were applied (Table 3).

**Table 3:** Multivariate Cox regression analysis for predictors of breast cancer‐specific survival according to the different methods of Ki67 assessment with different Ki67 cutoffs

Cutoff Scoring Method	10%			14%			20%			30%		
	HR	95% CI	*P*‐value	HR	95% CI	*P*‐value	HR	95% CI	*P*‐value	HR	95% CI	*P*‐value
Counting Ki67‐positive cells per 1000 invasive tumour cells	2.5	1.4–4.5	**0.002**	1.9	1.1–3.1	**0.040**	2.0	1.2–3.2	**0.007**	2.5	1.4–4.4	**0.001**
Average estimation within the hotspot	1.6	1.1–2.4	**0.018**	1.1	0.8–1.7	0.520	1.3	0.8–2.1	0.180	0.8	0.8–2.6	0.180
Average estimation in whole slide	0.9	0.5–1.6	0.810	1.5	0.8–2.5	0.190	1.0	0.6–1.6	0.900	0.3	0.3–1.2	0.170

HR, hazard ratio; CI, confidence interval.

Significant *P* values are in bold.

**Figure 4:**
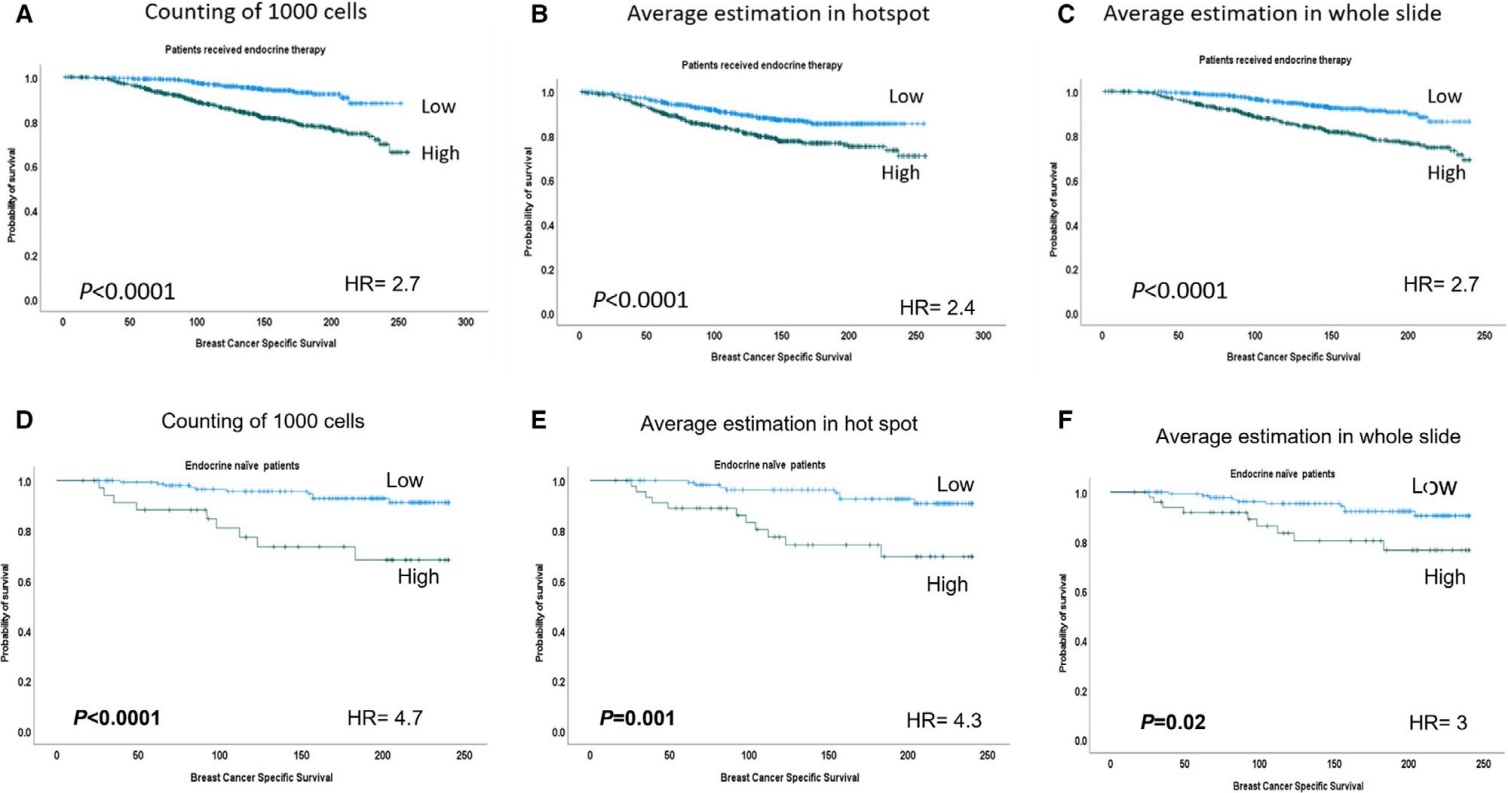
Kaplan–Meier plots show association of Ki67 expression levels in different scoring methods and breast cancer‐specific survival in patients, who received endocrine therapy using a 10% cutoff (**A–C**). Similar findings were observed in endocrine‐naïve patients using the same cutoff (**D–F**).

Regarding adjuvant therapy, there was a significant association between high Ki67 and poor outcome in patients who received endocrine therapy regardless of the method of scoring (Figure 4). However, in multivariate analysis the quantification of Ki67‐positive cells among 1000 tumour cells in the hotspot method showed a statistically significant prognostic value independent of the other two methods (Supplementary Table S3). In endocrine‐naive patients, there was also a significant association between high Ki67 scores and poor outcome using all methods. No association was observed between Ki67 and the outcome in patients treated with combined endocrine and chemotherapy.

**Figure 5:**
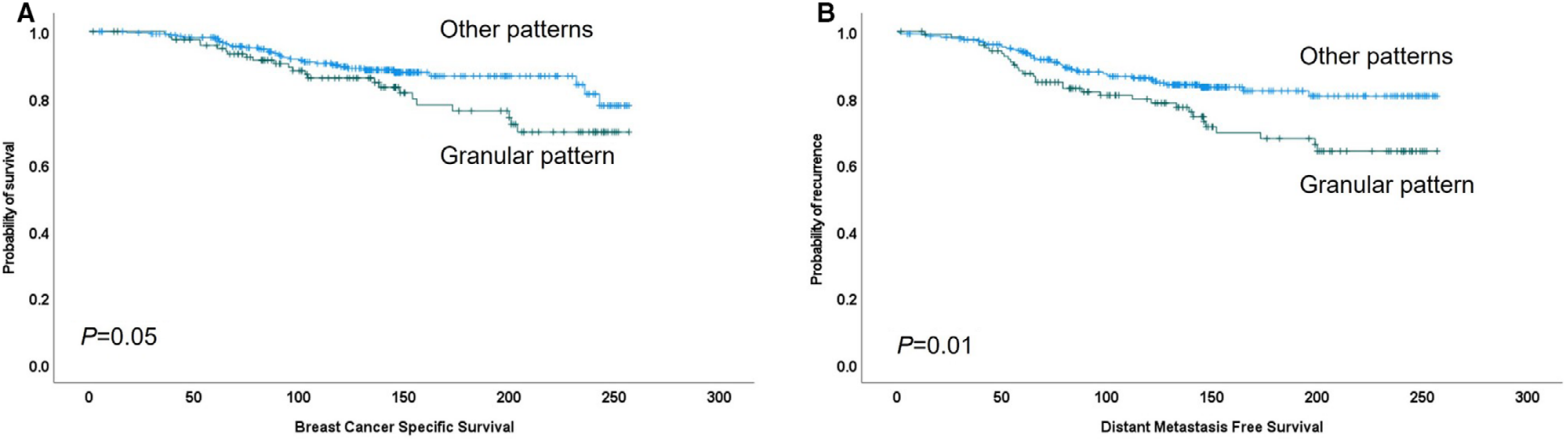
Kaplan–Meier plot shows the association between granularity pattern of Ki67 expression in luminal breast cancer and breast cancer‐specific survival (**A**) and distant metastasis‐specific survival (**B**).

There was a strong association between the Ki67 granular expression pattern and shorter DMFS (*P* = 0.009) and a trend of association with BCSS (Figure 5). Tumours that showed a high Ki67 level and more granular pattern showed the worst outcome with DMFS (*P* = 0.009) compared to low Ki67 expression with a homogeneous pattern (Supplementary Figure S2).

Neither H‐score nor Quick scores of Ki67 showed a significant association with patient outcome apart from an association with shorter DMFS, shown in cases with a higher Ki67 H‐score (Supplementary Figure S3).

### Clinical impact of Ki67 intratumoural heterogeneity on BC

**Figure 6:**
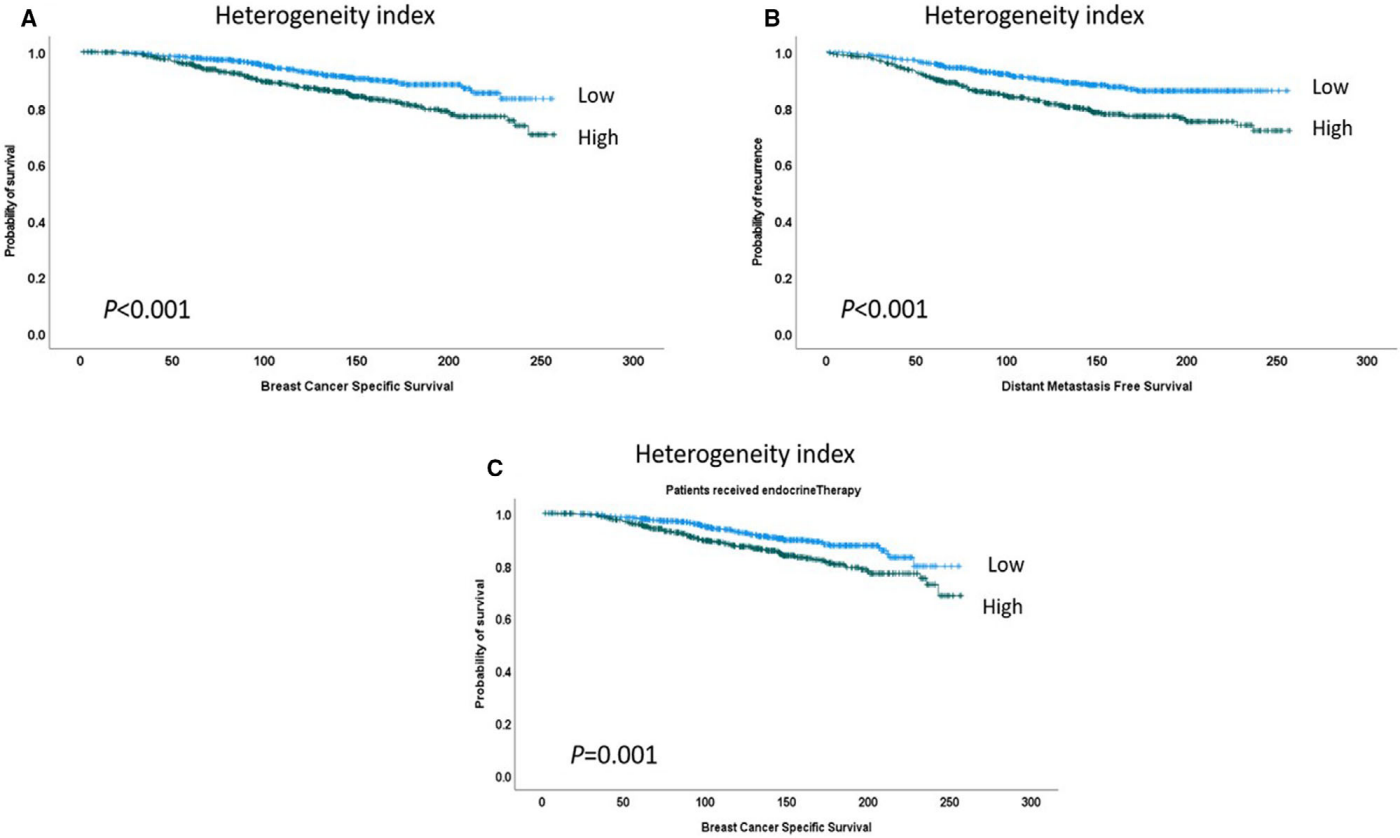
Kaplan–Meier plots show significant associations between Ki67 heterogeneity index and shorter breast cancer‐specific survival (**A**), and distant metastasis‐specific survival (**B**) and BCSS in patients who received endocrine therapy (**C**).

The mean Ki67 expression difference (heterogeneity index) between hotspot and whole slide was 2.8% (range 0–66%). There was a significant association between high heterogeneity index and larger tumour size, higher tumour grade, NST tumour type, presence of both LVI and lymph node metastasis, and poor prognostic NPI (Supplementary Table S4). Tumours with higher Ki67 expression had significantly higher heterogeneity values (*P* < 0.001) (Supplementary Figure S4). Additionally, a high heterogeneity index showed a strong association with shorter BCSS and DMFS (both *P* < 0.0001) in the whole cohort and when the analysis was confined to patients who received endocrine therapy (Figure 6).

## DISCUSSION

Although Ki67 is an essential indicator of cellular proliferation in malignancy and has a prognostic value in BC, there is no standard method yet to evaluate its level of expression.^11^ Several methods for Ki67 scoring have been proposed, including the IKWG that recently recommended standardised scoring with online application to score 100 nuclei either negative or positive in each field directed by the application and then record the “Weighted global score”, which is considered the Ki67 index. This method is tedious and requires routine digitalisation of the stained sections. In addition, the median required time to score a single case was 9 min, as the pathologist needs to review the Ki67‐stained slide and estimate the percent area with four categories; negligible, low, medium, or high Ki67 expression, then score 100 nuclei in each selected area in the whole slide and, finally, record the Ki67 index for that slide, which would be challenging to achieve in routine practice.^2^ Others adopted a simple 5‐grade scale to assess Ki67, which depends on visual estimation of the ratio between positive and negative cells. Although it was considered a fast and easy task, it led to a discrepancy of 26% of cases compared to the manual counting in the hotspot.^14^ The World Health Organization recommends visual counting of the positive Ki67 cells among at least 1000 invasive tumour cells; however, it was thought that the task may be a labour‐intensive task for pathologists.^8^, ^20^ To resolve this problem, automated counting by a computer software device might be a breakthrough; however, not all institutes can afford this due to a lack of the required infrastructure for pathology service digitalisation.^29^

In this study we compared three main methods for evaluating the percentage of Ki67 positivity in luminal (ER‐positive, HER2‐negative) BC, where Ki67 has a specific prognostic and predictive role. We also highlighted the clinical value of the staining pattern and intensities, which could reflect the underlying biology. When we initiated this study, we considered other published methods of Ki67 scoring; however, some were excluded due to the reported high level of discrepancy or for showing no or less significant association with patient outcome.^14^, ^15^, ^30^, ^31^ Moreover, in a previous unpublished study we assessed the average Ki67 in the whole slide by counting Ki67‐positive cells within 1000 cells in multiple randomly selected areas, regardless of the Ki67 hotspot area in a large number of cases (*n* = 610) as recommend by some authors.^15^ However, our results demonstrated a significant discrepancy in Ki67 scoring when compared with the other established methods, and it was not associated with patient outcome. Therefore, we concluded that counting 1000 cells in random areas does not represent the actual Ki67 scoring, and this method was not included in the current comparative study.

Although high Ki67 expression, regardless of assessment method, showed a significant association with parameters associated with aggressive tumour behaviour, including higher grade, larger tumour size, LVI, and lymph node invasion, the best association was related to the quantification method. This indicates that the hotspot has the highest proliferative pool, which drives tumour behaviour and counting of those cells represents the optimal method for Ki67 assessment. The quantification method was the longest, timewise, method compared to the other two methods; however, it was the most accurate method to predict patients` outcome and showed the highest interobserver concordance. This indicates that the same hotspot areas can be commonly picked by observers due to its specific criteria.

Although each of the methods used had a significant association with disease outcome, calculation of Ki67 percentage of positivity through counting the Ki67‐positive cells among 1000 invasive tumour cells within the hotspot showed the highest hazard ratio, the lowest *P* value, and showed independent prognostic value compared to the other two methods. Using common cutoff points for categorisation of Ki67 expression into low and high groups, only Ki67 with the quantification method showed consistent, significant association with poor outcome in a multivariate analysis, while the other two methods lost significance. It has been reported that a higher Ki67 value using the hotspot is strongly correlated with poor outcome and the determination of proliferation markers in BC should be standardised to hotspot counting, as it showed greater risk of shorter tumour‐free survival.^15^, ^32^ In addition, in a previous study, using digital image analysis for Ki67 scoring, quantification Ki67‐positive cells in hotspots was found to be superior to average Ki67 and even outweigh the mitotic count in the association with BC outcome.^33^ The IKWG recommends scoring the average Ki67, as this was based on the lack of sufficient evidence to use the “hotspot”^8^ and with the context of using core biopsy to assess Ki67,^2^ which may lack a representative hotspot for the tumour proliferation index due to intratumoural heterogeneity. Others claimed that the prognostic powers of Ki67 in luminal BC measured by either hotspot or average were similar.^12^ High Ki67 expression using all the previous scoring methods and had a strong association with poor outcome in patients who received endocrine therapy. However, high Ki67 expression using the quantification method had a stronger association with poor outcome in those groups of patients compared to the other two methods, which suggests that the accuracy of this method to predict therapy response and confirm that high proliferative tumours showed worse outcomes and those patients should be considered for more aggressive therapy, including chemotherapy. Also, it was found that Ki67 scoring using hotspot areas represents higher proliferation rates that could drive the biological behaviour of tumour.^13^

In patients who received both endocrine and chemotherapy, there was no association between high Ki67 and poor outcome, which indicate that patients with high Ki67 expression showed a better response and showed an outcome similar to patients with low Ki67. This suggests that adding chemotherapy to high Ki67 cases can improve the outcome.

We suggest using quantification in the hotspot method rather than average methods for being associated with the highest hazard risk. However, this method is time‐consuming. To avoid this limitation and achieve the most reliable and clinically applicable method, we tested utilizing the power of the quantification method in a simplified less time‐consuming method. The number of fields to count Ki67 depended on the cellularity of the tumour and the microscope field diameter. Consequently, pathologists scoring Ki67 in the clinical setting can calibrate their HPF and estimate the number of tumour cells per HPF in tumours with different cell density and assess the approximate number of HPFs that are required to count 1000 cells per case, without the actual cell count in every case. Then they can estimate the average percentage of Ki67‐positive cells in the 1000 cells, eyeballing without an actual count of the cells. Our results showed that this method reduced the scoring time (1–3 min per case) and achieved a high concordance rate with the actual counting of every case. This simplified method can be implemented in routine practice, but it needs training and retesting occasional cases for the actual count to ensure that scorers are within the accepted rate of estimation of the average cell count per HPFs. Although this method can by perceived by some as similar to the other two methods, it provides consistency and reproducibility and reduce the variation between cases with different cell density when scoring using fixed areas. In cases with a Ki67 score around the cutoff between high and low Ki67 categories, which may have therapeutic implications, detailed counting of 1000 cells within the hotspot may be considered to ensure reliability and accuracy.

In the current study, a homogeneous Ki67 staining pattern was prevalent in about 80% of luminal BC and was associated with a good outcome, while a granular pattern showed a strong association with DMFS and was an independent prognostic factor. This indicates that granular pattern could provide information about the patient's prognosis.

It has been shown that luminal BC is heterogeneous in terms of Ki67 expression, which is evident by the marked hotspot, and this heterogeneity is one of the obstacles that makes the standardisation of Ki67 interpretation difficult and decreases its reproducibility.^12^ In addition, in this study we found that Ki67 scores in hotspot versus average scores affect the categorisation of BC into the high versus low proliferation subgroups. Therefore, we calculated the difference between both scores and found a significant association between high heterogeneity index and parameters characteristic of aggressive tumour behaviour. Not only did the high Ki67 heterogeneity indices have a significant association with poor outcome in BC patients, but also it predicts poor response to endocrine therapy. Similarly, a previous study found a significant correlation between a higher Ki67 heterogeneity index and recurrence in luminal BC.^32^

Moreover, in our study the high heterogeneity index was significantly associated with tumours with high Ki67 expression, and this can explain the aggressive behaviour in tumours showing a high heterogeneity index in luminal BC.^32^

In conclusion, based on the previous findings, the quantification per 1000 cells method is considered the optimal method of Ki67 scoring, as it shows the highest hazard risk. Heterogeneity of Ki67 staining and patterns of its expression could give valuable information about the clinical outcome of BC.

## Ethical Approval and Consent to Participate

This work obtained ethics approval to use the human tissue samples by the North West – Greater Manchester Central Research Ethics Committee under the title; Nottingham Health Science Biobank (NHSB), reference number 15/NW/0685. Informed consent was obtained from all individuals and all samples were anonymised.

## Author Contributions

AL scored the cases and wrote the article draft, data analysis, and interpretation; MT helped in data analysis interpretation. AL, MT, NM, and AG agree with the article results and conclusions and critically reviewed the article. E.A. Rakha: conceived and planned the study, contributed to data interpretation, made critical revisions, and approved the final version.

## Funding

AL is supported by and funded by the Egyptian Ministry of Higher Education and Scientific Research.

## Conflicts of Interest

The authors declare that they have no conflicts of interest.

## Supporting information


**Appendix S1** Supporting information

## Data Availability

All data used in this study are archived and are available on reasonable request.
