# Supplementary material for: Ki67 assessment in invasive luminal breast cancer: a comparative study between different scoring methods
Source: Histopathology. 2022 Sep 19;81(6):786–98. doi: 10.1111/his.14781 (PMC9826086; doi:10.1111/his.14781)
Supplement: Supplementary file 1 — Appendix S1 Supporting information [file HIS-81-786-s001.docx]

**Supplementary Materials**

**Supplementary Table (1)** **Clinicopathological characteristics of the study cohort**

| Variables | No (%) |
| --- | --- |
| Age at diagnosis (years)  < 50  ≥ 50 | 390 (25)  1193 (75) |
| Menopausal state  Premenopausal  Post-menopausal | 452 (29)  1131 (71) |
| Tumour size (cm)  ≤ 2  > 2 | 1054 (67)  529 (33) |
| Histologic tumour grade  Grade1  Grade 2  Grade 3 | 358 (23)  812 (51)  413 (26) |
| Histologic tumour types  No special type (NST)  Lobular  Other special types  NST mixed | 836 (53)  187 (12)  334 (21)  226 (14) |
| Lymph node metastasis  Absent  Present | 1098 (69)  485 (31) |
| Lymhovascular invasion  Absent  Present | 1246 (79)  337 (21) |
| Nottingham prognostic index  Good prognostic group  Moderate prognostic group  Poor prognostic group | 765 (48)  721(46)  97 (6) |

**Supplementary Table 2: Low versus high Ki67 expression groups with different methods of scoring using multiple cut-offs**

*Note that higher percentages of the tumours that showed high proliferation index were consistently noticed in the counting of Ki67 positive cells/1000 tumour cells using different cut-offs

| Cut-off* | 10%  (n, %) | 14%  (n, %) | 20%  (n, %) | 30%  (n, %) |
| --- | --- | --- | --- | --- |
| Counting per 1000 cells  Low  High | 713 (45)  870 (55) | 926 (59)  657 (41) | 1196 (76)  387 (23) | 1416 (90)  167 (10) |
| Average estimation within the hotspot  Low  High | 900 (57)  683 (43) | 1079 (68)  504 (32) | 1312 (82)  271 (18) | 1452 (92)  131 (8) |
| Average estimation in whole slide  Low  High | 835 (53)  748 (47) | 1052 (67)  531 (33) | 1314 (83)  269 (17) | 491(94)  92 (6) |

**Supplementary table 3: Multivariate Cox regression analysis for predictors of breast cancer-specific survival according to the different methods of Ki67 assessment with different Ki67 cut-offs on patients who received endocrine therapy**

| **Cut-off** **Scoring**  **Method** | **10%** | | | **14%** | | | **20%** | | | **30%** | | |
| --- | --- | --- | --- | --- | --- | --- | --- | --- | --- | --- | --- | --- |
|  | Hazard ratio | 95% CI | P-value | Hazard ratio | 95% CI | P-value | Hazard ratio | 95% CI | P-value | Hazard ratio | 95% CI | P-value |
| Counting Ki67 positive cells per 1000 invasive tumour cells | 2.8 | 1.5-5.2 | **0.002** | 1.8 | 0.9-3.2 | 0.06 | 1.8 | 1.1-3.1 | **0.02** | 2.5 | 1.4-4.3 | **0.001** |
| Average estimation within the hotspot | 1.4 | 0.97-2.2 | **0.073** | 1.2 | 0.76-1.7 | 0.56 | 1.3 | 0.83-2 | 0.3 | 1.4 | 0.8-2.5 | 0.3 |
| Average estimation in whole slide | 0.93 | 0.5-1.6 | 0.059 | 1.3 | 0.75-2.4 | 0.19 | 1.8 | 0.6-1.7 | 0.97 | 0.5 | 0.3-1.1 | 0.085 |

CI, confidence interval

**Significant p values are in bold**

| Variables | Number (%) | **Heterogeneity index** | | X^2^  *P*-value |
| --- | --- | --- | --- | --- |
|  |  | **Low (n, %)** | **High (n, %)** |  |
| **Age (years)**  <50  ≥50 | 390 (25)  1193 (75) | 192(49)  675 (57) | 198 (51)  518 (43) | 6.4  **0.01** |
| **Menopausal state**  Premenopausal  Post-menopausal | 452 (29)  1131 (71) | 235 (52)  632 (56) | 217 (48)  499 (44) | 1.97  0.16 |
| **Tumour size (cm)**  ≤2  >2 | 1054 (67)  529 (33) | 623 (59)  244 (46) | 431 (41)  285 (54) | 23.9  **<0.0001** |
| **Tumour grade**  1  2  3 | 358 (23)  812 (51)  413 (26) | 263 (74)  460 (57)  144 (40) | 95 (27)  352 (43)  269 (65) | 117.7  **<0.0001** |
| **Histologic tumour types**  No special type (NST)  Lobular  Other special types  NST mixed | 836 (53)  187 (12)  334 (21)  226 (14) | 411 (49.2)  104 (56)  225 (67)  127 (56) | 425 (51)  83 (44)  109 (33)  99 (44) | 32.2  **<0.0001** |
| **Lymph node invasion**  Absent  Present | 1098 (69)  485 (31) | 623 (57)  244 (50) | 475 (43)  241 (50) | 5.6  **0.01** |
| **Lymphovascular invasion**  Absent  Present | 1246 (79)  337 (21) | 714 (57)  153 (45) | 532 (43)  184 (55) | 15.2  **<0.0001** |
| **Nottingham prognostic index**  Good prognostic group  Moderate prognostic group  Poor prognostic group | 765 (48)  721 (46)  97 (6) | 500 (65)  334 (46)  33 (34) | 265 (35)  387 (54)  64 (66) | 72.3  **<0.0001** |

**Supplementary table 4: Relationship between** **Ki-67 heterogeneity index and clinicopathological parameters**


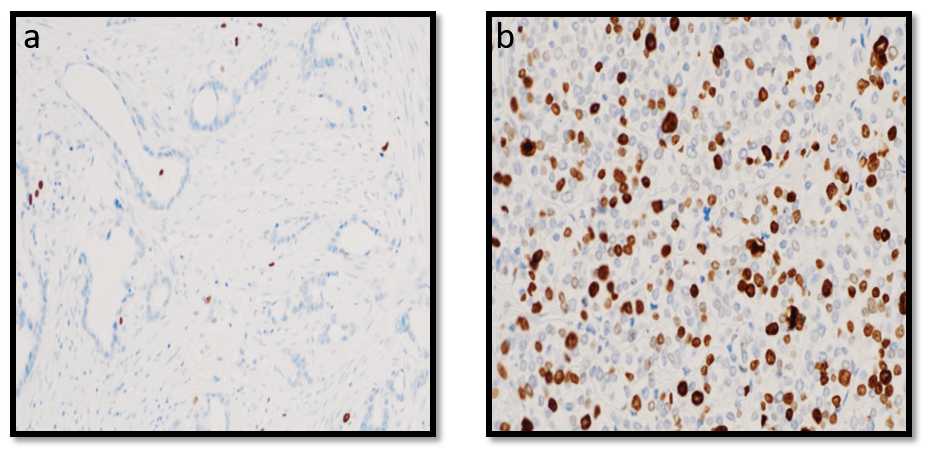


Supplementary figure 1: a) a case of BC shows very low expression of Ki67 while b) another case shows high expression of Ki67.


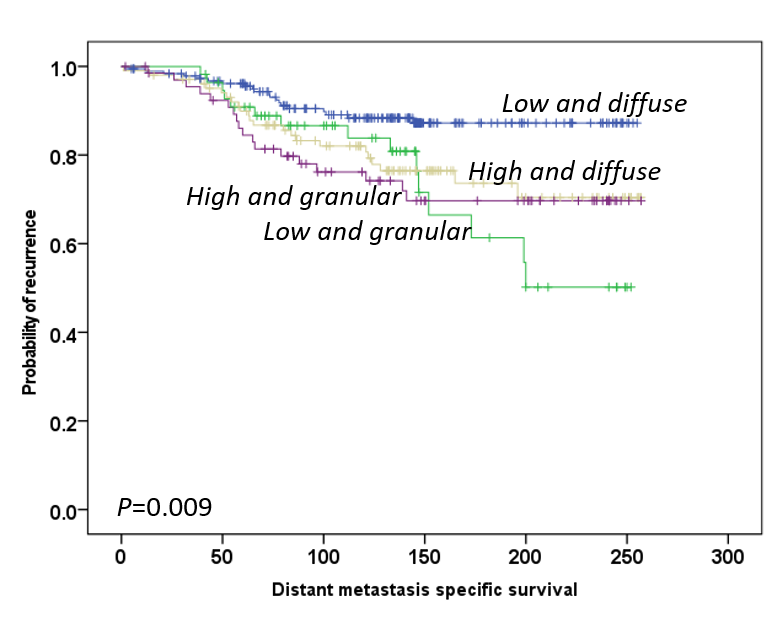


Supplementary Figure 2: (a) Kaplan–Meier plot shows the association of high Ki67 expression with granular pattern and shorter distant metastasis free survival.


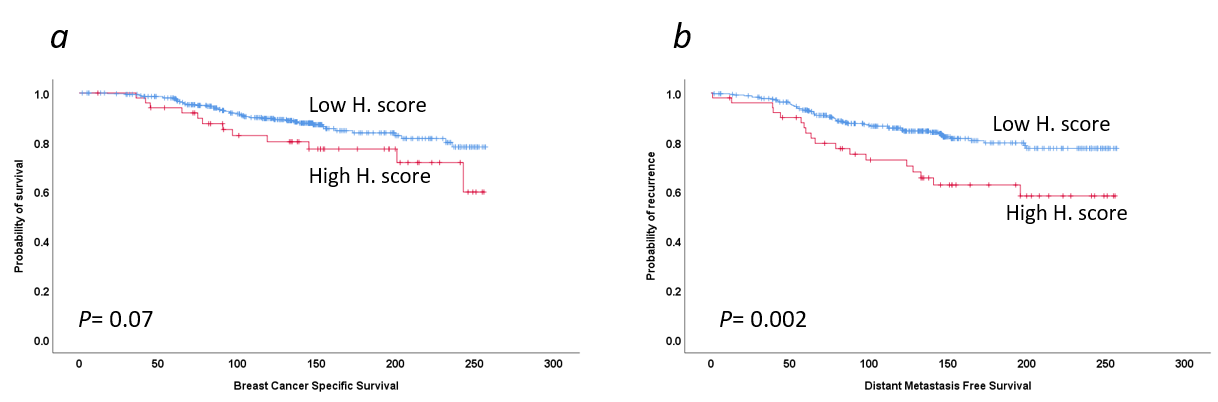


Supplementary Figure 3: (a) Kaplan–Meier plot shows borderline association between H-score of Ki67 expression and poor BCSS while strong association was observed between high score and DMFS (b).


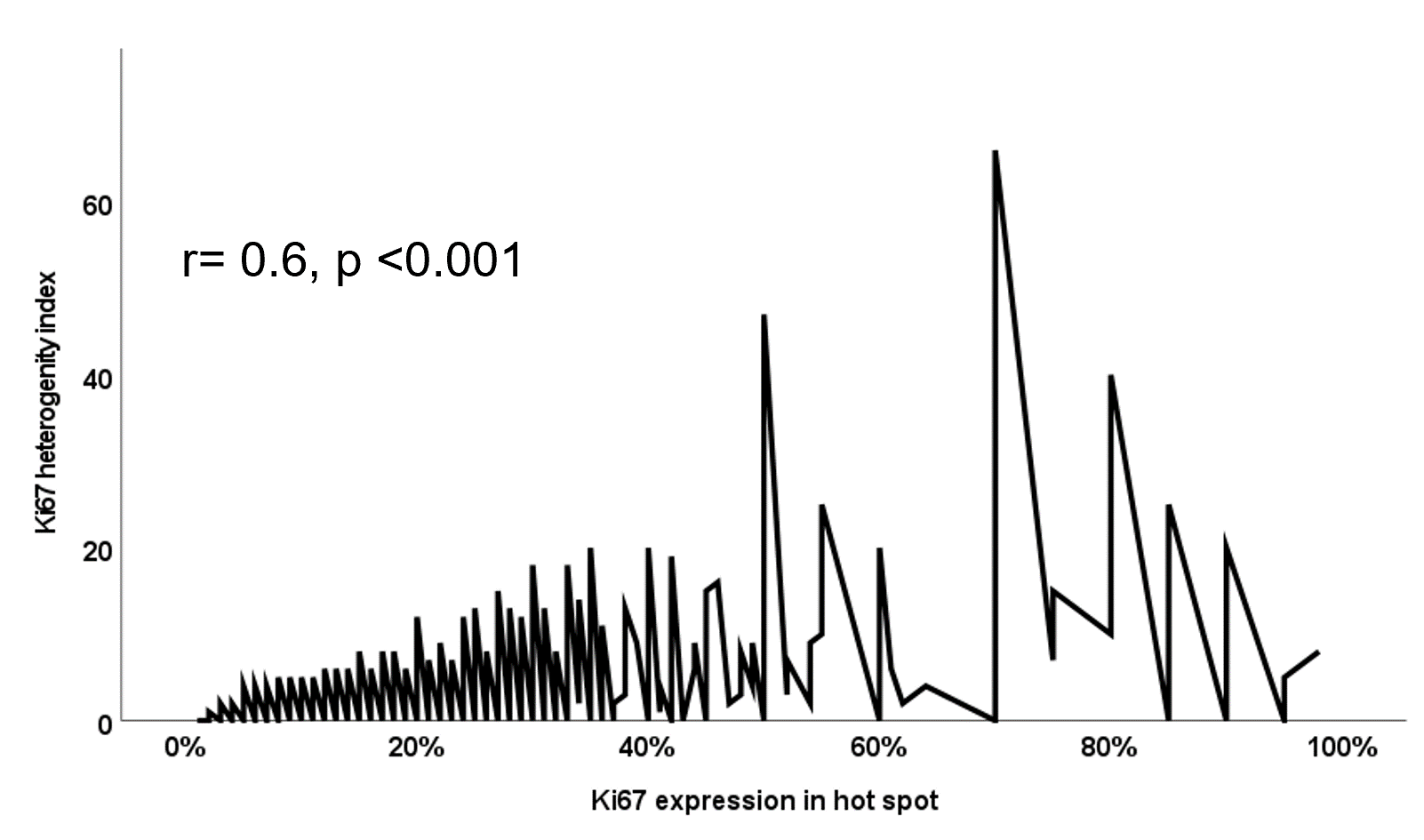


Supplementary Figure 4: Line graph shows the correlation between Ki67 heterogeneity index (difference between Ki67 expression in hot spot and average) and the Ki67 expression levels in hot spot.
